# Incidentally Detected Cystic Pheochromocytoma on Computed Tomography in a Patient Presenting With a Urinary Tract Infection: A Report of a Rare Case

**DOI:** 10.7759/cureus.105947

**Published:** 2026-03-26

**Authors:** Chetana Ratnaparkhi, Avinash Dhok, Bheekam Kurmi, Nisha B Meshramm

**Affiliations:** 1 Department of Radiology, All India Institute of Medical Sciences (AIIMS) Nagpur, Nagpur, IND; 2 Department of Radiodiagnosis and Interventional Radiology, All India Institute of Medical Sciences (AIIMS) Nagpur, Nagpur, IND; 3 Department of Radiodiagnosis, All India Institute of Medical Sciences (AIIMS) Nagpur, Nagpur, IND; 4 Department of Pathology, All India Institute of Medical Sciences (AIIMS) Nagpur, Nagpur, IND

**Keywords:** computed tomography, cystic pheochromocytoma, incidental, rare case report, urinary tract infection

## Abstract

Pheochromocytomas are rare catecholamine-secreting neuroendocrine tumors originating from the adrenal medulla, presenting with classic symptoms of headache, diaphoresis, and palpitations due to paroxysmal hypertension. Generally, pheochromocytomas are solid or sometimes solid with cystic changes. Purely cystic pheochromocytoma is very uncommon, with only a limited number of cases reported. We present the case of a 42-year-old male with cystic pheochromocytoma who presented with symptoms of urinary tract infection and, on imaging, was found to have a left adrenal purely cystic lesion. On imaging, a cystic lesion with an enhancing wall, showing no solid component, calcification, hemorrhage, or necrosis, was noted in the left suprarenal region. On this, the possibility of pheochromocytoma was kept. However, his biochemical markers were normal. The patient underwent an adrenalectomy with excision of the lesion, and histopathology and immunohistochemistry confirmed it as a cystic pheochromocytoma. A purely cystic pheochromocytoma needs consideration as one of the diagnoses in cystic adrenal lesions, particularly in a patient with unusual clinical features, and imaging is suggestive of a purely cystic adrenal lesion.

## Introduction

Pheochromocytomas are rare neuroendocrine neoplasms of the adrenal medulla and sympathetic ganglia, causing catecholamine hypersecretion [1]. Most adrenal lesions are incidentalomas. Approximately 4-22% of all adrenal incidentalomas are cystic [2]. Cystic adrenal lesions are uncommon, with an incidence of 0.064-0.18% on autopsy [2].

The majority of adrenal cysts are asymptomatic and nonfunctioning [3]. Typically, pheochromocytomas are solid tumors that may show necrosis or cystic change. The purely cystic variety of pheochromocytoma is very rare, with a limited number of cases reported in the literature [4]. Differentiating these tumors from adrenal cysts and pseudocysts is essential for further management.

Here, we present a rare case of a cystic adrenal lesion with a non-specific clinical presentation. Imaging revealed a cystic lesion in the left suprarenal region without a solid component, and septations were noted. Pheochromocytoma was confirmed on histopathology and immunohistochemistry.

## Case presentation

A 42-year-old male patient presented with complaints of burning in micturition and dull aching left flank pain, radiating to the groin for 15 days. It is not associated with vomiting, hematuria, or lithuria. The patient was a known hypertensive and diabetic. The patient gave a history of one episode of a hypertensive crisis five months ago. The patient also had a cerebrovascular accident (CVA) one year ago, from which he has fully recovered. The patient gave a history of recurrent renal calculi. He underwent percutaneous transluminal coronary angioplasty (PTCA) of the left circumflex artery one year ago. The patient gave no history of purplish stria, cushingoid features, or stigma of neurocutaneous syndrome. Table 1 shows the patient’s laboratory investigation.

**Table 1:** Details of laboratory investigations

Parameter	Patient’s Result	Normal Reference Value
Procalcitonin	16.51 ng/ml	<20 ng/ml
Blood Urea	9 mg/dl	15–39 mg/dl
Serum Creatinine	0.9 mg/dl	0.6–1.3 mg/dl
Hemoglobin	8.4 g/dl	13–17 g/dl
Hematocrit	16%	20–60%
Serum Sodium	130 mmol/l	135–145 mmol/l
Serum Potassium	4.8 mmol/l	3.5–5.5 mmol/l
Serum Phosphorus	5 mg/dl	2.5–4.5 mg/dl
Serum Magnesium	1.3 mg/dl	1.6–2.6 mg/dl
Fasting Plasma Glucose	91.1 mg/dl	70–105 mg/dl
Post Prandial Plasma Glucose	107.4 mg/dl	<140 mg/dl
Plasma Normetanephrine	123 pg/ml	<148 pg/ml
Plasma Metanephrine	42 pg/ml	<57 pg/ml
24 hrs Urine Levels of Normetanephrine	198 mcg	75–375 mcg
24 hrs Urine Levels of Metanephrine	77 mcg	24–96 mcg

The patient’s hemoglobin was low, with low hematocrit values. His blood urea, serum sodium, and magnesium were low. Serum phosphorus was slightly raised. Serum procalcitonin level was raised. The plasma and urinary metanephrine and normetanephrine levels were within normal range.

**Figure 1:**
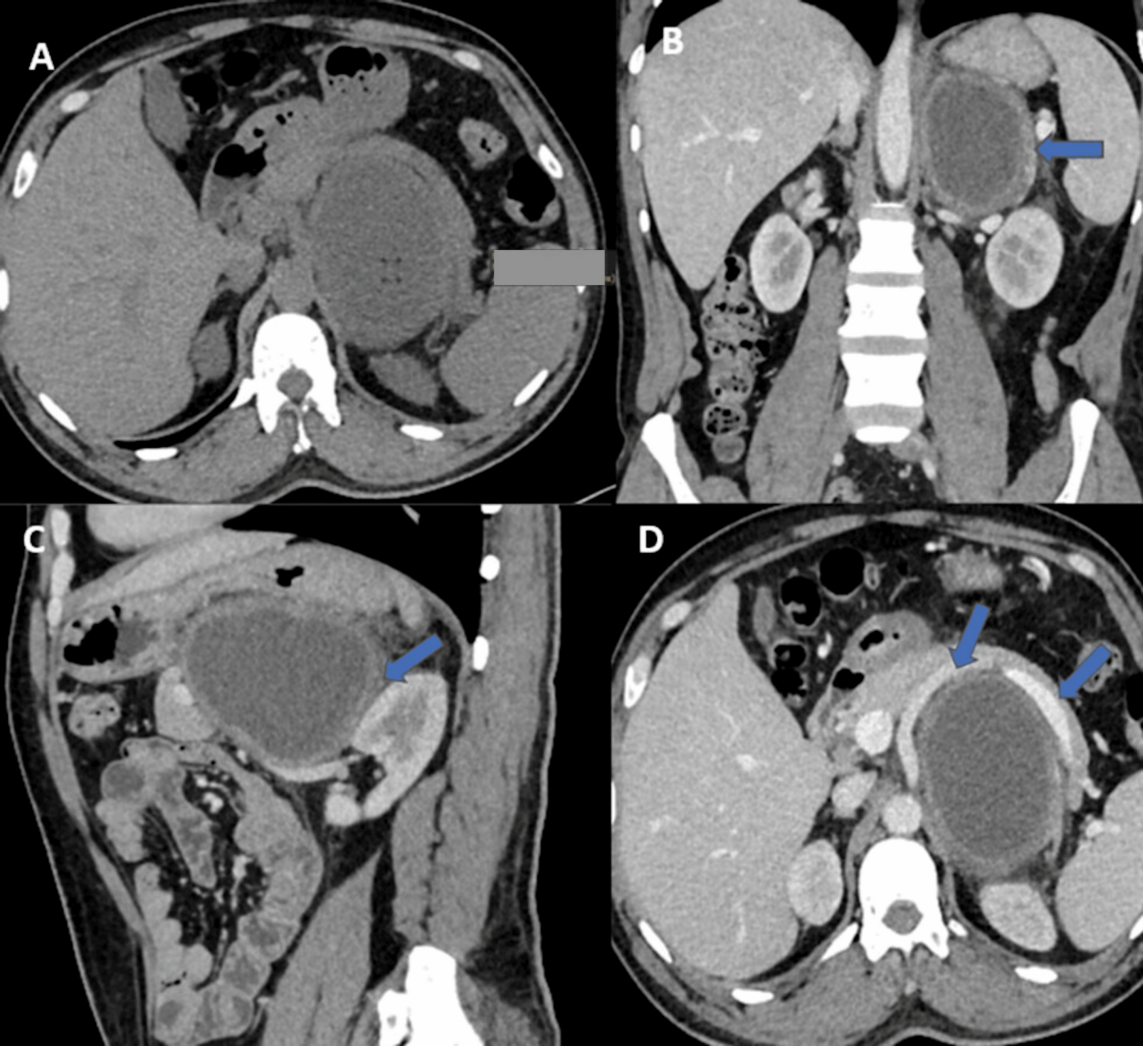
CT images of the patient (A) Non-contrast, axial image shows a fluid attenuation lesion in the left suprarenal location; (B) post-contrast, coronal CT image showing a cystic lesion with peripheral wall enhancement (arrow); (C) post-contrast, sagittal CT image showing the lesion is superior to the left kidney with maintained fat planes; (D) post-contrast, axial CT image showing anterior displacement of the left renal vessels by the lesion (arrows). CT: computed tomography

On ultrasonography (USG), a hypoechoic lesion in the left suprarenal region was seen. The left adrenal gland was not seen separately from the lesion. In contrast-enhanced computed tomography (CECT) of the abdomen, a well-defined, thick-walled, peripherally enhancing, cystic lesion was in the left suprarenal region; however, the left suprarenal gland was not seen separately (Figure 1).

The lesion does not show calcification, septations, a solid component, or hemorrhage. The lesion showed maintained fat planes with the adjacent organs. Antero-inferiorly, it was compressing the left renal vein and postero-inferiorly abutting the left renal artery without any vascular invasion/thrombosis.

Based on these findings, the possibility of cystic pheochromocytoma was given. An adrenal cyst (endothelial type) was kept as a differential diagnosis (less likely).

**Figure 2:**
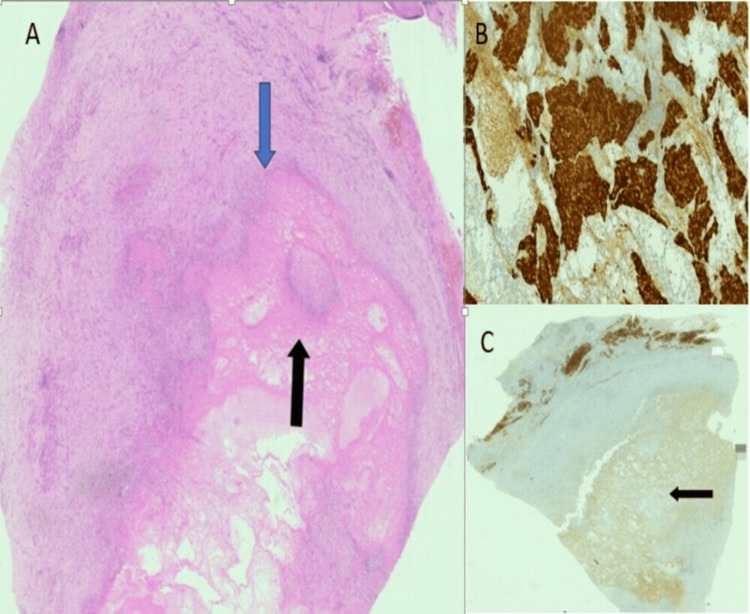
Histopathology images (A) H&E/4×: cystic lesion with central necrotic tumor (black arrow) and surrounding fibrocollagenous wall (blue arrow); (B) 20×: chromogranin IHC: strongly positive in necrotic tumor (brown cytoplasmic stain); (C) 20×: synaptophysin IHC: weakly positive in necrotic tumor (faint brown cytoplasmic stain, black arrow), peripherally preserved cortical tissue. IHC: immunohistochemistry; H&E: hematoxylin and eosin

The patient underwent left adrenalectomy with excision of the mass. Histopathology revealed a necrotic adrenal tumor with a trabecular, nested pattern and lymphoplasmacytic infiltrate. Immunohistochemistry showed tumor cells positive for chromogranin and synaptophysin, confirming the diagnosis of pheochromocytoma (Figure 2).

## Discussion

Pheochromocytomas are rare neuroendocrine, catecholamine-secreting tumors from chromaffin cells of the adrenal medulla or sympathetic ganglia [5]. Nearly 80-85% are from the adrenal gland and referred to as pheochromocytoma, while around 15-20% originate from sympathetic ganglia and are labeled as paragangliomas. It can occur sporadically or as a part of a familial syndrome, e.g., Von Hippel-Lindau disease or neurofibromatosis [6].

These tumors are mainly seen in individuals aged 20-50 years old [5]. One study by Andreoni et al. found that the cystic variety of pheochromocytoma is more common in females [7]. Clinically, it can present with the Menard triad consisting of headache, palpitation, and sweating associated with hypertension due to catecholamine hypersecretion [8]. However, a few patients with pheochromocytoma can be normotensive and asymptomatic [9].

Pheochromocytomas can be solid or solid cystic, which can be due to hemorrhage or necrosis. The prevalence of purely cystic pheochromocytomas is not well known, and very few cases are reported in the literature. Patients with cystic pheochromocytomas are likely to be asymptomatic or may present with non-specific symptoms and signs, and also have negative biochemical markers compared to those with solid tumors [7,10].

In the present case, the patient had typical symptoms, but the plasma and urinary metanephrine and normetanephrine values were within the normal range; the reason for this is unclear. One possible explanation is that the patient may be having essential hypertension and not the one related to increased catecholamine secretion. Our patient had lithuria and a urinary tract infection. He also had a past history of PTCA and CVA, which appears to be unrelated to the recent diagnosis of pheochromocytoma. The patient reported a history of dull, aching pain on the left side, which may be due to the mass effect caused by the large suprarenal lesion.

Imaging is important for the morphological assessment of the lesions. On USG, pheochromocytomas appear as well-defined, oval to round lesions with variable appearances, ranging from solid to mixed solid-cystic to purely cystic [11]. The size of these lesions ranges from 1 to 15 cm [12]. The small lesions are more or less homogeneous, while large lesions show heterogeneity due to hemorrhage or necrosis, giving a cystic appearance.

When imaging a patient with an adrenal lesion on CT, the adrenal protocol is preferred. On CT, pheochromocytomas appear as a well-defined lesion in the suprarenal location, which may be solid or cystic, showing significant vascularity. The most common differential diagnosis for pheochromocytoma is an adenoma. The Hounsfield unit (HU) value of pheochromocytoma is >10 HU, while that of adenoma is <10 HU, and pheochromocytoma shows delayed washout, while adenoma shows rapid washout on contrast CT. However, some pheochromocytomas can show rapid washout, as seen in an adenoma [13].

Cystic adrenal lesions can be categorized on CT as uncomplicated, indeterminate, or complicated. Uncomplicated lesions are less than 6 cm in size and show homogeneous water attenuation with a thin wall. Indeterminate lesions are larger, show increased attenuation, and have a thick wall, while complicated lesions show high attenuation and are heterogeneous with hemorrhage or calcifications [14]. Typical CT appearance of the purely cystic variety of pheochromocytoma is a well-defined lesion of near-water attenuation showing thick wall enhancement, which may or may not show septa. CT is useful for involvement/invasion of adjacent organs, multiplicity, vascular invasion, and preoperative planning.

MRI is not a routinely used imaging modality for evaluating pheochromocytoma. However, it is useful in indeterminate adrenal lesions, pregnant patients, pediatric cases, and patients with iodinated contrast allergies in CT [7]. On MRI, the T1 and T2 appearances of pheochromocytoma depend on the content, i.e., solid, cystic, hemorrhage, and necrosis. The purely cystic form of pheochromocytoma shows T2 hyperintensity and T1 hypointensity without any intralesional enhancement. For pheochromocytoma, commonly used molecular imaging tests include 123I-MIBG scintigraphy, 18F-FDG or 18F-DOPA PET/CT, and somatostatin receptor imaging [15].

Approximately 10% of pheochromocytomas are malignant. However, we did not find any data regarding purely cystic pheochromocytomas showing metastasis. In the present case, no metastasis was seen.

Histopathology is the gold standard for confirmation of the cystic variety of pheochromocytoma. Histopathologically, the Pheochromocytoma of the Adrenal Gland Scaled Score (PASS) is used to determine the risk of malignancy in the tumors [16]. The closest differential diagnosis for purely cystic adrenal pheochromocytoma is an endothelial lymphatic cyst or pseudocyst. A cystic pheochromocytoma should be considered when the lesion demonstrates a relatively thick wall and persistent contrast enhancement.

The gold standard treatment for pheochromocytoma is surgical resection. Preoperative pharmacological management is aimed at reducing the intraoperative complications and perioperative mortality.

In the present case, the patient underwent left adrenalectomy with excision of the mass, with an uneventful intraoperative and postoperative status. The patient has been advised to follow up after six months.

## Conclusions

In the evaluation of an adrenal cystic lesion, a cystic pheochromocytoma should be considered when the lesion demonstrates a relatively thick wall (with or without internal septations) and persistent post-contrast wall enhancement, especially when the patient is asymptomatic with negative biochemical markers. A multidisciplinary approach plays a key role in dealing with cystic adrenal lesions to avoid an intraoperative hypertensive crisis.
